# Treadmill running improves hindlimb arteriolar endothelial function in type 1 diabetic mice as visualized by X-ray microangiography

**DOI:** 10.1186/s12933-015-0217-0

**Published:** 2015-05-13

**Authors:** Takashi Sonobe, Hirotsugu Tsuchimochi, Daryl O. Schwenke, James T. Pearson, Mikiyasu Shirai

**Affiliations:** Department of Cardiac Physiology, National Cerebral and Cardiovascular Center Research Institute, Suita, Osaka Japan; Department of Physiology-Heart Otago, University of Otago, Dunedin, New Zealand; Department of Physiology, and Monash Biomedical Imaging Facility, Monash University, Melbourne, Australia; Australian Synchrotron, Clayton, Australia

**Keywords:** *in vivo*, Vascular imaging, Limb blood flow distribution, Exercise training

## Abstract

**Background:**

Vascular function is impaired in patients with diabetes, however diabetic vascular dysfunction is ameliorated by exercise training. We aimed to clarify which hindlimb arterial segments are affected by treadmill running in the hindlimbs of streptozocin-induced type 1 diabetic mice *in vivo*.

**Methods:**

Mice were divided into 3 groups; healthy control, diabetic control, and diabetic-running groups. The exercise regimen was performed by treadmill level running mice for 60 min/day, for 4 weeks. Thereafter, we examined the vascular response to systemic acetylcholine administration in the left hindlimb of anesthetized-ventilated mice using either 1) X-ray microangiography to visualize the arteries or 2) ultrasonic flowmetry to record the femoral arterial blood flow.

**Results:**

X-ray imaging clearly visualized the hindlimb arterial network (~70-250 μm diameter). The vasodilator response to acetylcholine was significantly attenuated locally in the arterioles <100 μm diameter in the diabetic group of mice compared to the control group of mice. Post-acetylcholine administration, all groups showed an increase in hindlimb vascular conductance, but the diabetic mice showed the smallest increase. Overall, compared to the diabetic mice, the treadmill-running mice exhibited a significant enhancement of the vasodilator response within the arterioles with diabetes-induced vasodilator dysfunction.

**Conclusions:**

Diabetes impaired acetylcholine-induced vasodilator function locally in the arteries <100 μm diameter and decreased hindlimb vascular conductance responded to acetylcholine, while regular treadmill running significantly ameliorated the impaired vasodilator function, and enhanced the decreased conductance in the diabetic mice.

## Background

Micro- and macro-vascular function is impaired in both patients with type 1 and 2 diabetes mellitus. It is well accepted that the endothelial dysfunction has an impact contributing to the failure of peripheral circulatory regulation in hyperglycemia [1]. Using an experimental type 1 diabetic animal model, earlier studies have investigated the impairment of peripheral vascular function with isolated mesenteric [2] and femoral arteries [3]. In the long term, this endothelium-dependent peripheral vascular dysfunction results in severe hindlimb ischemia, which has obvious clinical significance [4-6]. Considering this problem, studies focusing on hindlimb peripheral arterial function have been the target of therapeutic approaches with the aim of preventing diabetic peripheral arterial disease [7].

It has also been reported that regular physical exercise can ameliorate diabetic-induced endothelial dysfunction in clinical [8] and experimental animal studies [9,10]. However, there is a paucity of direct evidence concerning the contribution of exercise training to the amelioration of endothelial function in the diabetic hindlimb vasculature *in vivo*. No direct measurement of vascular diameter has been carried out to determine which hindlimb arterial segments actually benefit from the effect of exercise training. Methodological constraints associated with problems of observing *in vivo* vascular flow, such as the lack of transparency of tissues, have made it difficult to assess vascular reactivity directly within the hindlimb, as with other intravital organs (heart, lung and brain). Indeed, for this reason the relationship between blood flow and arterial pressure, i.e. vascular conductance has been used to estimate vascular reactivity in these organs [11-13].

In a series of studies using *in vivo* X-ray microangiography, we and others have demonstrated visualization of the vessel network of intravital organs and investigated functional changes of vessels in the rodents heart [14,15], lungs [16,17], and brain [18-21]. Microangiography has the ability to visualize the complex vessel network from large conduit arteries to the small branches of resistance vessels within individual organs. Moreover, it is possible to identify the vessel branching order associated with regional vascular dysfunction. In this study, we elucidated which vascular segments endothelium dependent vasodilator function was improved by exercise training in type 1 diabetic mice. We directly measured the changes in vascular inner diameter in response to acetylcholine infusion (endothelium dependent vasodilator) in the hindlimb arterial network from the conduit arteries to the arterioles (~70-250 μm diameter) using *in vivo* X-ray microangiography. We also measured arterial blood pressure and femoral blood flow to assess hindlimb vascular conductance.

## Methods

### Animals

All experiments were conducted in accordance with the Guide for the Care and Use of Laboratory Animals, 8th Edition (National Research Council (US) Institute for Laboratory Animal Research). The study was approved by the Animal Subjects Committee of the National Cerebral and Cardiovascular Center, Japan.

Fifty two male C57BL/6NCr mice (7 week old, Japan-SLC, Hamamatsu, Japan) were randomly assigned into 3 groups, a sedentary control (Ctrl; n = 18), a sedentary diabetic (Stz; n = 18), and treadmill running diabetic (Exe; n = 16) groups. Stz and Exe groups of animals received a single intraperitoneal dose of streptozocin (200 mg/kg, Sigma, Tokyo, Japan) in 0.1 M citrate buffer, pH 4.5, to induce type 1 diabetes. All mice were on a 12 h light/dark cycle and were provided with food and water ad libitum. Two weeks after the streptozocin injection, blood glucose was measured using a glucose monitoring system in conscious mice (Precision Xceed, Abbott, Tokyo, Japan) via the tail vein following 8 h of fasting. The onset of diabetes was confirmed by fasting blood glucose concentration over 200 mg/dl and body weight loss. Six weeks after streptozocin injection, all mice underwent terminal experiments. Blood glucose was measured again on the day prior to the terminal experiment.

### Treadmill running

Two weeks after the streptozocin injection, the running regime for the Exe group mice commenced. Treadmill running consisted of progressive stages of level running for 60 min, 5 days/week for 4 weeks on a rodent treadmill (MK-680, Muromachi Kikai, Tokyo, Japan). Running was started at a speed of 8 m/min in the first week; the maximal speed in a session was raised by 2 m/min in every week. According to previous reports [22,23], the level of oxygen consumption is estimated to be 76 % of VO_2_ max during 12 m/min of treadmill running in healthy adult C57BL6 mouse. The exercise intensity of our training protocol was considered to be in the range of moderate to hard, and this level of physical activity was expected to achieve similar benefits to that reported in type 2 diabetic patients [24]. Before and after each session of treadmill running, 5 min of running at a speed of 6 m/min was performed as a warming-up and cooling-down. Only animals which ran steadily on the treadmill were included in the study. All mice were used for experiments within 3 days after the last session of treadmill running.

### Surgical preparation

Mice were anesthetized by intraperitoneal injection of urethane (600 mg/kg) and α-chloralose (60 mg/kg). Throughout the experimental protocol, body temperature was maintained at 36-37°C using a thermostatically controlled heating pad and/or heating lamp. The mouse was securely restrained on an acrylic board in a supine position during the surgical procedure.

The trachea was cannulated, and the lungs were ventilated with a mouse ventilator (6 μl/g tidal volume and 180 breaths/min; Minivent Type 845; Hugo Sachs. Elektronik–Harvard Apparatus, March-Hugstetten, Germany). The inspired gas was enriched with oxygen. A Silastic tube (0.012” inner diameter, Dow Corning, Midland, MI) was cannulated into the right jugular vein for acetylcholine administration. A tapered PE-50 catheter filled with heparinized saline (50 units/ml), was inserted into the right carotid artery to record arterial blood pressure via a pressure transducer. The pressure signals were relayed to a BP Amp (ML117; AD Instruments Japan Inc., Nagoya, Japan) and then continuously sampled at 1 kHz with a PowerLab system (AD Instruments Japan Inc., Nagoya, Japan) and recorded on a computer using Chart software (AD Instruments Japan Inc., Nagoya, Japan) throughout the experiment. Heart rate was derived from the arterial systolic peaks and mean arterial pressure was calculated online. As a vehicle, lactate Ringer’s solution was administered intravenously via the right jugular vein to maintain body fluids (0.25 μl/g/min). Separate groups of mice were used in the following surgical procedures and experiments for either microangiography or blood flow measurement.

### X-ray digital subtraction microangiography

Twenty three mice (Ctrl, 8; Stz, 8; Exe, 7) were assigned for microangiography experiments. Following the general surgical preparation, an additional tapered PE-50 catheter was retrogradely inserted into the right femoral artery for contrast medium injection. Microangiography was conducted with a purpose built angiographic system (MFX-80HK, Hitex, Osaka, Japan) consisting of an open type 1 μm microfocus X-ray source (L9191, Hamamatsu Photonics, Hamamatsu, Japan) and a 50/100 mm (2”/4”) dual mode X-ray image intensifier (E5877JCD1-2 N, Toshiba, Tokyo, Japan) operating at 70 kV and 30 μA current. Microangiography sequences were recorded digitally at the rate of 30 frames/s as a series of 640 × 480 pixel bitmap image files. The mouse was positioned on the plate above the X-ray source so that the left hindlimb was positioned in center of the X-ray detector field of view. After verifying whether the imaging field comprised the left femoral arterial vessels, iodinated contrast medium (Iomeron 350, Eisai, Tokyo, Japan) was injected intra-arterially as a bolus (80 ~ 90 μl at a speed of 8 ml/min) into the abdominal aortic bifurcation with an injection pump (PHD-2000, Harvard Apparatus, Holliston, MA). X-ray image acquisition was initiated from one second before iodine injection, and continued for five seconds.

**Fig. 1:**
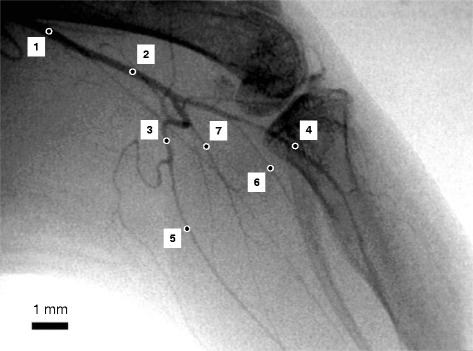
Original X-ray angiogram of the left hindlimb vasculature in a control mouse. Femoral arteries were visualized by bolus intra-arterial injection of iodinated contrast agent. Femur and tibia are also evident. Closed-circles placed on the arteries indicate the points, which were measured for inner diameter. The numbers are corresponding to the arteries averaged for vessel diameters in Table 1. Scale bar = 1 mm

Image analysis was performed as previously described [17]. The image analysis program ImageJ (National Institutes of Health) was used to evaluate the vessel inner diameter. To enhance images, a temporal digital subtraction operation was performed for flat-field correction using summation results of sixteen consecutive frames acquired before contrast agent injection. The summation image taken before injection was subtracted from a summation image taken after injection to eliminate the superimposed background structure. Seven of the visible and identifiable vessels were used for inner diameter measurement (Fig. 1). Inner diameter of the vessels was directly measured using Straight-Line-Selections tool of ImageJ. A resolution test chart of known linear separations was subsequently used as a reference for calculating vessel diameter. The measured vessels were categorized according to the size of inner diameter: conduit arteries, over 100 μm arteries, and under 100 μm arterioles. In each category, the % changes in vessel diameter for individual segments during acetylcholine were averaged.

### Ultrasonic blood flow measurement

Twenty nine mice (Ctrl, 10; Stz, 10; Exe, 9) were assigned for blood flow experiments. Following the general surgical preparation, the left femoral artery was carefully isolated from surrounding tissue and an ultrasound flow probe (0.5 mm, 0.5PSB, Transonic Systems Inc., Ithaca, NY) was placed around the artery, 3–5 mm distal to the inguinal ligament. Femoral arterial blood flow was measured by a transit-time flowmeter (TS420, Transonic Systems Inc., Ithaca, NY). The flow signals were continuously monitored and recorded simultaneously with blood pressure signals. Absolute value of femoral blood flow was normalized by body weight of the animal. Hindlimb vascular conductance was determined by dividing femoral blood flow by mean arterial pressure.

### General experimental protocol

Endothelium-dependent vasodilatory responses were tested in both microangiography and blood flow experiments with a continuous intravenous acetylcholine infusion. After the surgical procedure, mice were given at least 10 min of recovery period, which was sufficient time for all cardiovascular variables to stabilize, and then baseline angiography and/or hemodynamic data were obtained with a continuous infusion of vehicle solution via the jugular vein (0.25 μl/g/min). After the baseline data acquisition, acetylcholine was continuously infused at a dose of 0.1 μg/kg/min for 10 min, and data acquisition was performed. Data acquisition and imaging was repeated with higher dose of 1.0 μg/kg/min.

### Statistical analysis

All statistical analyses were conducted with GraphPad Prism 5 (GraphPad Software Inc., La Jolla, CA). All results are presented as means ± SE. Body weight, fasting blood glucose, and baseline hemodynamic parameters among the three groups were compared by one-way ANOVA followed by a Newman-Keuls test. The original, averaged vessel inner diameter was compared by one-way ANOVA followed by a Newman-Keuls test among the three groups. The % change of vessel diameter and % change of vascular conductance were compared by one-way ANOVA followed by a Newman-Keul’s test. Differences were considered significant at P < 0.05.

## Results

### X-ray microangiography

**Fig. 2:**
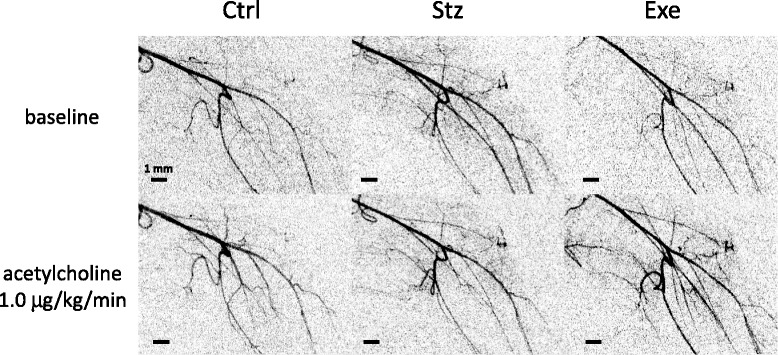
Representative subtracted angiograms of the hindlimb vasculature in control (Ctrl), diabetic (Stz), and treadmill running diabetic (Exe) mice during baseline (upper panels) and following administration of acetylcholine (1.0 μg/kg/min, lower). The original image was subtracted by background image captured before contrast agent injection to enhance the edge of vessels. Scale bar = 1 mm

A typical angiographic image of a control mouse is presented in Fig. 1. Femoral arteries were visualized with the overlaying femur and tibia, and this original image was subtracted by background image to enhance the edge of vessels (Fig. 2). Closed circles shown in the Fig. 1 indicate the seven vessels identified for inner diameter measurement, since those vessels were visualized in almost all images. During the baseline condition the femoral conduit was greater than 200 μm and other conduit arteries, small arteries-arterioles ranged between 70–250 μm diameters in all groups (Table 1). The baseline diameters were not statistically different in the three groups.

**Table 1:** Averaged baseline vessel diameters (μm) obtained from the angiograms

	Conduit artery	Over 100 μm arteries			Under 100 μm arteries		
Vessle #	#1	#2	#3	#4	#5	#6	#7
Ctrl	222 ± 12 (8)	177 ± 8 (8)	141 ± 5 (8)	126 ± 10 (8)	100 ± 5 (8)	87 ± 4 (8)	86 ± 5 (6)
Stz	207 ± 10 (8)	194 ± 9 (8)	135 ± 8 (8)	133 ± 6 (8)	92 ± 6 (8)	84 ± 5 (8)	79 ± 3 (6)
Exe	234 ± 9 (7)	192 ± 15 (7)	122 ± 6 (7)	113 ± 6 (7)	97 ± 5 (7)	76 ± 9 (7)	73 ± 5 (7)

The vessel number (#) is corresponding to the numbers depicted in Fig. 1. Numbers in parentheses represent the number of vessels used to determine the group average of the vessel diameters. All values are expressed as mean ± SE

**Fig. 3:**
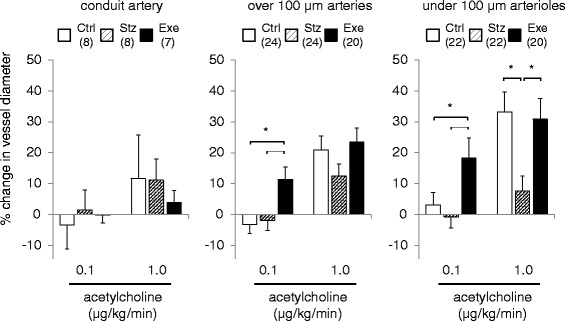
Percent changes in vessel inner diameter in control (Ctrl: n = 8), diabetic (Stz: n = 8), and treadmill running diabetic (Exe: n = 7) mice during infusion of 0.1 and 1.0 μg/kg/min acetylcholine, categorized by vessel class, 1) conduit (popliteal) artery, 2) over 100 μm arteries, and 3) under 100 μm arterioles. Data in parentheses represent the number of vessels which were averaged. Values are expressed as mean ± SE. *P < 0.05

Acetylcholine infusion caused typical vasodilatation in all groups (Fig. 2). Figure 3 shows the mean % change in vessel diameters across the 3 different size orders during continuous acetylcholine infusion. In the control group of mice, acetylcholine-induced vasodilation was mainly observed at 1.0 μg/kg/min dose. The better effect was found in the arteries > 100 μm diameter (20.9 ± 4.5 % of dilation from baseline diameter, P < 0.01) and the arterioles < 100 μm diameter (33.2 ± 6.5 % of dilation from baseline diameter, P < 0.01), while not in conduit artery (11.6 ± 14.1 %). The diabetic group of mice showed a significant attenuation of the vasodilation with acetylcholine (1.0 μg/kg/min) in the arterioles < 100 μm diameter (7.6 ± 4.9 % of dilation from baseline, P < 0.05 compared to Ctrl), but not in the conduit arteries and the small arteries over 100 μm diameter. The treadmill-running mice showed that treadmill running acts mainly on the arterioles <100 μm diameter to enhance the vasodilation responses to both doses of acetylcholine (0.1 and 1.0 μg/kg/min) and significantly improved the attenuated vasodilation with acetylcholine (1.0 μg/kg/min) to almost the same level of dilation as that in the control (30.9 ± 6.6 % of dilation, P < 0.05 compared to Stz).

**Fig. 4:**
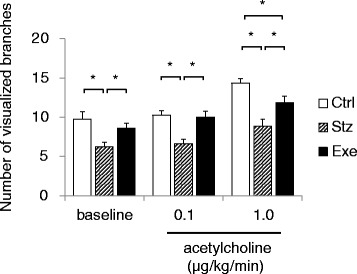
Number of visualized (contrast agent delivered) femoral arterial branches in control (Ctrl: n = 8), diabetic (Stz: n = 8), and treadmill running diabetic (Exe: n = 7) mice during baseline, infusion of 0.1 and 1.0 μg/kg/min acetylcholine. Values are expressed as mean ± SE. *P < 0.05

The number of visualized vessel branches was counted in all images (Fig. 4). In the baseline condition, the average number of branches was 9.8 ± 0.9 in the control group. The number of branches was significantly lower in the diabetic group (6.3 ± 0.6, P < 0.05 vs Ctrl), while it was greater following treadmill running (8.6 ± 0.6, P < 0.05 vs Stz). During acetylcholine infusion at 1.0 μg/kg/min, the number of branches was significantly increased (P < 0.05) in all the groups of mice, but not during 0.1 μg/kg/min. The numbers of branches in the diabetic group were significantly fewer than those in the control during any dose of acetylcholine infusion (P < 0.05 vs Ctrl) and were significantly higher following treadmill running (P < 0.05, vs Exe), although the number of branches in the treadmill-running group was significantly smaller (P < 0.05) than the control during 1.0 μg/kg/min acetylcholine infusion.

Table 2 shows animal metabolic characteristics and baseline hemodynamic parameters for the groups of mice for the angiography experiments. In the control, body weight and blood glucose were 27 ± 1 g and 131 ± 7 mg/dl, respectively. Both diabetic and treadmill-running groups, which were given streptozocin, showed a significant loss of body weight (Stz, 18 ± 1 g; Exe, 17 ± 1 g; P < 0.05 vs Ctrl) and high fasting blood glucose (Stz, 412 ± 22 mg/dl; Exe, 408 ± 13 mg/dl; P < 0.05 vs Ctrl) at the end of the 6 week period. Resting heart rate and mean arterial pressure were 549 ± 24 beats/min and 80 ± 2 mmHg, respectively in the control group, and these were not different among the groups.

**Table 2:** Animal characteristics and baseline values obtained from the mice in the microangiography protocol

	Body weight	Blood glucose	Heart rate	Mean arterial pressure
	(g)	(mg/dl)	(beats/min)	(mmHg)
Ctrl (8)	27 ± 1	130 ± 6	540 ± 22	80 ± 2
Stz (8)	18 ± 1 *	400 ± 23 *	534 ± 10	87 ± 3
Exe (7)	17 ± 1 *	408 ± 13 *	557 ± 16	88 ± 3

Numbers in parentheses represent the number of mice. All values are expressed as mean ± SE. *P < 0.05 vs Ctrl

### Blood flow measurement

**Fig. 5:**
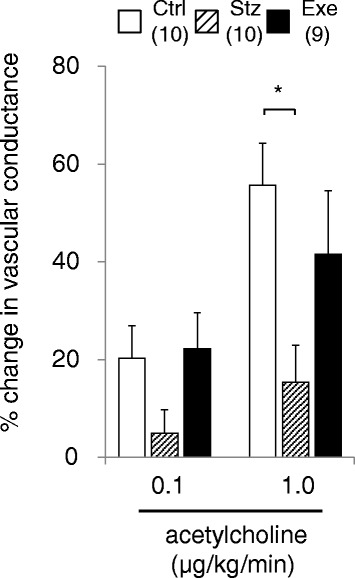
Acetylcholine-induced % change in hindlimb vascular conductance in control (Ctrl: n = 10), diabetic (Stz: n = 10), and treadmill running diabetic (Exe: n = 9) mice during infusion of 0.1 and 1.0 μg/kg/min of acetylcholine. Values are expressed as mean ± SE. *P < 0.05

Figure 5 indicates the changes of estimated hindlimb vascular conductance in response to continuous acetylcholine infusion. Similar to the angiography vascular diameter changes, acetylcholine increased peripheral vascular conductance in the control group, dose-dependently (increased 20.3 ± 6.7 % at 0.1 μg/kg/min, and 55.7 ± 8.5 % at 1.0 μg/kg/min acetylcholine from baseline). The vascular conductance response was significantly attenuated in the diabetic group at 1.0 μg/kg/min of acetylcholine (15.4 ± 7.6%, P < 0.05 compared to Ctrl), but not at 0.1 μg/kg/min, as seen in the attenuated vasodilator response of the arterioles <100 μm. Treadmill running mostly restored the attenuated response of vascular conductance to the same level as that in the control, although no significant difference was found between diabetic and treadmill-running groups.

Table 3 shows the animal characteristics and baseline hemodynamic parameters for each group of mice in the experiments that assessed blood flow using the transit-time ultrasound recordings. In the control group, body weight and blood glucose were 28 ± 1 g and 121 ± 7 mg/dl, respectively. Both diabetic and treadmill-running groups demonstrated body weight loss (Stz, 19 ± 1 g; Exe, 20 ± 1 g) and high blood glucose (Stz, 389 ± 21 mg/dl; Exe, 391 ± 37 mg/dl), similar to the mice in the angiography experiment. Resting heart rate and mean arterial pressure were 461 ± 16 beats/min and 67 ± 3 mmHg respectively, and resting femoral blood flow and vascular conductance were 490 ± 39 μl/min/100 g BW and 7.4 ± 0.6 μl/min/mmHg/100 g BW in the control group. These baseline values were not different among the groups.

**Table 3:** Animal characteristics and baseline values obtained from the mice in the flowmetry protocol

	Body weight	Blood glucose	Heart rate	Mean arterial pressure	Femoral blood flow	Hindlimb vascular conductance
	(g)	(mg/dl)	(beats/min)	(mmHg)	(μl/min/100 g BW)	(μl/min/mmHg/100 g BW)
Ctrl (10)	28 ± 1	121 ± 7	464 ± 16	67 ± 3	490 ± 39	7.4 ± 0.6
Stz (10)	19 ± 1 *	389 ± 21 *	491 ± 19	68 ± 2	627 ± 84	9.4 ± 1.4
Exe (9)	20 ± 1 *	391 ± 37 *	496 ± 17	71 ± 2	603 ± 85	8.6 ± 1.3

Numbers in parentheses represent the number of animals. All values are expressed as mean ± SE. *P < 0.05 vs Ctrl

## Discussion

The present study demonstrated that regular treadmill running ameliorates the reduction in acetylcholine-induced hindlimb blood distribution in streptozocin-induced type 1 diabetic mice. *In vivo* microangiography visualized hindlimb vasculature and acetylcholine infusion-provoked vasodilation in anesthetized mice. Of note is that the vasodilator response in the arteries smaller than 100 μm, which was greater than that in the larger arteries, was significantly attenuated in diabetic mice, and treadmill running restored the attenuated response to almost the same level as that in the control group. Moreover, the response of hindlimb vascular conductance with acetylcholine was also reduced in diabetic mice, and the reduced response was mostly restored by treadmill running.

### Digital subtraction hindlimb microangiography

In this study, we used a laboratory X-ray system with a 1 μm microfocus X-ray source, and investigated the hindlimb peripheral vascular function in anesthetized mice. The hindlimb vessels, from large conduit femoral artery (diameters over 200 μm) to small artery-arterioles (diameters less than 100 μm), were clearly visualized with a conventional angiographic method, using bolus intra-arterial injection of iodinated contrast agent during each image acquisition (Figs. 1 and 2). In the last decade, angiograms of intra-organ microvasculature in the rodents heart [14,15], lungs [16,17], and brain [19-21] were successfully obtained by using in-vivo microangiography with synchrotron radiation as an X-ray source [25]. Some of the earlier studies using laboratory X-ray imaging sources also demonstrated the brain microvasculature in small animals [18,26]. These *in vivo* microangiographic techniques allow us to obtain an angiogram with acceptable spatial and time resolution for dynamic, real-time vascular analysis. Compared to the earlier laboratory X-ray imaging system with a spatial resolution of approximately 50 μm [18], our microfocus X-ray system has a spatial resolution of ~10 μm and is acceptable for measuring the dynamic diameter changes of ~50 μm arterioles in mice.

We found that a vessel diameter-dependent difference in the impairment of vasodilator responses to acetylcholine between large to medium conduit arteries and small arteries-arterioles in mice hindlimb *in vivo*. It has been suggested based on studies using the perfused mesenteric arterial bed as a small resistance artery, that the vasodilator response to acetylcholine is markedly impaired in diabetic rats [27]. The microangiography results demonstrated local impairment of acetylcholine-induced endothelium dependent vasodilation within the arterioles <100 μm, rather than large arteries, these finding were consistence with the previous findings obtained by perfused preparation. Meanwhile, impaired vasodilation of the arterioles was significantly recovered in the treadmill-running diabetic mice compared to the sedentary diabetic mice. It has been proposed that diabetic peripheral vascular dysfunction is ‘reversible’ by the positive effect of exercise training [8,9,28], which is consistent with our own results. Since our data did not demonstrate the typical exercise training effects, such like decrease in baseline heart rate, blood glucose control, and prevented loss of body weight, it cannot be excluded other stress responses, which could affect to the vascular functions.

Our data in Fig. 3 demonstrated that there was no major vasodilation, thus no functional impairment was found in arteries of the diabetic mice compared to the control mice when 0.1 μg/kg/min of acetylcholine was administered. Interestingly, significant increases in vessel diameter were found in small arteries but not in conduit artery from the treadmill-running diabetic mice during 0.1 μg/kg/min of acetylcholine administration. Since it has been suggested that exercise training increases sensitivity to nitric oxide in hindlimb resistance vessels [29-31], our data may suggest that regular treadmill running increases sensitivity to acetylcholine even though the vasodilative function was impaired by the diabetic condition. Meanwhile Zguira et al. reported that diabetes-induced endothelial dysfunction in rat aorta was not restored by intense physical training [32]. This finding is partly consistent with our data, which demonstrated no improvement of acetylcholine-induced vasodilation in femoral conduit artery, while we found that acetylcholine-induced vasodilatory function was ameliorated in the small arteries (<100 μm) in treadmill-running diabetic mice. Therefore the data obtained from X-ray imaging would be potentially powerful tool to investigate small/resistance arterial functions *in vivo*.

We also found that the number of visualized vessels was decreased in the diabetic mouse in baseline and during acetylcholine infusion (Fig. 4). Most of the small arteries, which are less than 100 μm and usually found in the angiogram of hindlimb of healthy mouse, were not depicted by angiography in the diabetic mouse. In other words, the small vessels in diabetic mice were not well perfused by contrast agent or the contrast agent-delivered to vessels that were small enough to be below the range of the spatial resolution of our X-ray microangiography system. This result, however, does not necessarily imply that there was arteriosclerotic narrowing in the arterioles or avascular area in the examined hindlimb, but possibly suggests that there was lowered blood flow at the small vessel level (might include capillaries), as shown by the reduced red blood cell flow in the skeletal muscle of type 1 diabetic rats [33]. In addition to the significantly improved acetylcholine-induced vasodilation reported here, we found that the number of visualized vessels in the hindlimb was restored by the regular treadmill running. Here also, endothelium-dependent mechanisms might contribute to the restoration of perfusion after treadmill running, but further studies will be required to reveal details of these mechanisms.

### Blood flow measurement and its limitations

Our data for baseline femoral blood flow in the control mice was consistent with that previously reported [34]. Since the number of visualized vessel branches was reduced in the diabetic group of mice (Fig. 4), we hypothesized that the absolute value of hindlimb blood flow would be significantly lower in diabetic mice than that of normal mice in the control group. However, the absolute value of resting femoral arterial blood flow was not significantly changed in the diabetic group of mice in comparison to the control, but the mean 12% lower flow is consistent in magnitude with the ~7% smaller conduit artery caliber we observed with microangiography. Similarly, the previous paper from Copp et al. [35] also demonstrated that the resting total hindlimb blood flow was not different between the control and the diabetic animals. In keeping with that study, the baseline blood flow, normalized by the animal’s body weight, was likely increased in the diabetic group of mice due to the loss of body weight, though there were no statistical differences among the groups.

Hindlimb vascular conductance, which in many laboratories is routinely estimated by femoral blood flow divided by mean arterial pressure, was also found to be depressed in diabetic mice and intermediate between diabetic and controls in the treadmill-running diabetic mice. While these findings appear to reject our hypothesis on first impression, we consider this disparity in the two approaches to detect blood flow changes is due to an important limitation of the ultrasonic flow probe method. Since the smallest available flow probe for direct quantification of flow velocity is only suitable for placement around the largest conduit vessel in the mouse hindlimb, the femoral artery, it measures total flow into the hindlimb but not necessarily perfusion of the skeletal muscle in the limb. In Fig. 2 the passage of iodine contrast agent shows that less iodine and therefore less blood flow, passes through the main second and third order branches of the tibial arteries in the diabetic mice, compared to either the control or the treadmill-running groups. This suggests the possibility that more of the blood is shunted in the diabetic mouse hindlimb through the saphenous artery distally without perfusing the gastrocnemius muscle. This final point is borne out by the significantly lower visualized vessel number in the diabetic mice. Hence, visualization of blood flow distribution to skeletal muscle via the resistance vessels is considered to be an advantage of the microangiographic approach which can be implemented in any laboratory with a similar commercial X-ray microfocus source.

### Limitation of exercise training on streptozocin-induced diabetic mice

Physical activity is considered as an important non-pharmacological strategy in the management of hyperglycemia not only in type 2 diabetic but also in type 1 diabetic patients [36]. The drug-induced type 1 diabetes animal model is widely used in studies of diabetic vascular response, and in present study, we tried to visualize the functions of vessels, which chronically exposed to high blood glucose level using the type 1 diabetic model mice. Although the regular treadmill running improved peripheral vascular function in this study, the treadmill running protocol did not ameliorate the loss of body weight and elevated blood glucose level in streptozocin induced type 1 diabetic mice. It is possible that the running protocol was not appropriate for the type 1 diabetic mice to maintain basal blood glucose level and body weight in terms of intensity and duration of running regimen. Further investigation will be required to explore the optimal protocol, along with the measurements, e.g. skeletal muscle citrate synthase activity test and glucose tolerance test, to validate the effects of exercise training.

Daily training thus increased their muscle activity, but did not restore body weight to levels similar to healthy animals. These results are in agreement with previous data reporting lack of improvement in type 1 diabetic animal’s body weight [37-39]. Since clinical studies have also failed to show the benefit of exercise training on glucose control in type 1 diabetic patients [40], the beneficial effects on glycemic control by exercise training may be limited without insulin administration [36]. Further study is needed to strengthen our evidence obtained from this study with combined therapy applying both exercise and insulin injection in type 1 diabetic animal model. In addition, use of other diabetic animal models such as diet-induced obesity and genetically altered diabetic mice will provide us further information.

## Conclusions

Diabetes impaired the acetylcholine-induced vasodilator function locally in the arteries less than 100 μm diameter and decreased hindlimb vascular conductance responded to acetylcholine, while regular treadmill running significantly improved the locally impaired vasodilator function and enhanced the decreased vascular conductance in diabetic mice. These findings provide evidence that exercise training has the potential to ameliorate the diabetes-induced endothelial vasodilator dysfunction chiefly in the arterioles (resistance vessels) governing hindlimb blood perfusion distribution.

## References

[1] Kamata K, Miyata N, Kasuya Y (1989). Impairment of endothelium-dependent relaxation and changes in levels of cyclic GMP in aorta from streptozotocin-induced diabetic rats. Br J Pharmacol.

[2] Fukao M, Hattori Y, Kanno M, Sakuma I, Kitabatake A (1997). Alterations in endothelium-dependent hyperpolarization and relaxation in mesenteric arteries from streptozotocin-induced diabetic rats. Br J Pharmacol.

[3] Shi Y, Ku DD, Man RY, Vanhoutte PM (2006). Augmented endothelium-derived hyperpolarizing factor-mediated relaxations attenuate endothelial dysfunction in femoral and mesenteric, but not in carotid arteries from type I diabetic rats. J Pharmacol Exp Ther.

[4] Cade WT (2008). Diabetes-related microvascular and macrovascular diseases in the physical therapy setting. Phys Ther.

[5] Hadi HA, Suwaidi JA (2007). Endothelial dysfunction in diabetes mellitus. Vasc Health Risk Manag.

[6] Potenza MA, Gagliardi S, Nacci C, Carratu MR, Montagnani M (2009). Endothelial dysfunction in diabetes: from mechanisms to therapeutic targets. Curr Med Chem.

[7] Delbin MA, Davel AP, Couto GK, de Araujo GG, Rossoni LV, Antunes E (2012). Interaction between advanced glycation end products formation and vascular responses in femoral and coronary arteries from exercised diabetic rats. PLoS One.

[8] Fuchsjager-Mayrl G, Pleiner J, Wiesinger GF, Sieder AE, Quittan M, Nuhr MJ (2002). Exercise training improves vascular endothelial function in patients with type 1 diabetes. Diabetes Care.

[9] Chakraphan D, Sridulyakul P, Thipakorn B, Bunnag S, Huxley VH, Patumraj S (2005). Attenuation of endothelial dysfunction by exercise training in STZ-induced diabetic rats. Clin Hemorheol Microcirc.

[10] Jasperse JL, Laughlin MH (2006). Endothelial function and exercise training: evidence from studies using animal models. Med Sci Sports Exerc.

[11] Arbin V, Claperon N, Fournie-Zaluski MC, Roques BP, Peyroux J (2000). Effects of combined neutral endopeptidase 24–11 and angiotensin-converting enzyme inhibition on femoral vascular conductance in streptozotocin-induced diabetic rats. Br J Pharmacol.

[12] Kiff RJ, Gardiner SM, Compton AM, Bennett T (1991). Selective impairment of hindquarters vasodilator responses to bradykinin in conscious Wistar rats with streptozotocin-induced diabetes mellitus. Br J Pharmacol.

[13] Kiff RJ, Gardiner SM, Compton AM, Bennett T (1991). The effects of endothelin-1 and NG-nitro-L-arginine methyl ester on regional haemodynamics in conscious rats with streptozotocin-induced diabetes mellitus. Br J Pharmacol.

[14] Jenkins MJ, Edgley AJ, Sonobe T, Umetani K, Schwenke DO, Fujii Y (2012). Dynamic synchrotron imaging of diabetic rat coronary microcirculation in vivo. Arterioscler Thromb Vasc Biol.

[15] Pearson JT, Jenkins MJ, Edgley AJ, Sonobe T, Joshi M, Waddingham MT (2013). Acute Rho-kinase inhibition improves coronary dysfunction in vivo, in the early diabetic microcirculation. Cardiovasc Diabetol.

[16] Schwenke DO, Pearson JT, Umetani K, Kangawa K, Shirai M (2007). Imaging of the pulmonary circulation in the closed-chest rat using synchrotron radiation microangiography. J Appl Physiol.

[17] Sonobe T, Schwenke DO, Pearson JT, Yoshimoto M, Fujii Y, Umetani K (2011). Imaging of the closed-chest mouse pulmonary circulation using synchrotron radiation microangiography. J Appl Physiol.

[18] Figueiredo G, Boll H, Kramer M, Groden C, Brockmann MA (2012). In vivo X-ray digital subtraction and CT angiography of the murine cerebrovasculature using an intra-arterial route of contrast injection. AJNR Am J Neuroradiol.

[19] Liu P, Sun J, Zhao J, Liu X, Gu X, Li J (2010). Microvascular imaging using synchrotron radiation. J Synchrotron Radiat.

[20] Shirai M, Schwenke DO, Eppel GA, Evans RG, Edgley AJ, Tsuchimochi H (2009). Synchrotron-based angiography for investigation of the regulation of vasomotor function in the microcirculation in vivo. Clin Exp Pharmacol Physiol.

[21] Kidoguchi K, Tamaki M, Mizobe T, Koyama J, Kondoh T, Kohmura E (2006). In vivo X-ray angiography in the mouse brain using synchrotron radiation. Stroke.

[22] Schefer V, Talan MI (1996). Oxygen consumption in adult and AGED C57BL/6 J mice during acute treadmill exercise of different intensity. Exp Gerontol.

[23] Fernando P, Bonen A, Hoffman-Goetz L (1993). Predicting submaximal oxygen consumption during treadmill running in mice. Can J Physiol Pharmacol.

[24] Colberg SR, Albright AL, Blissmer BJ, Braun B, Chasan-Taber L, Fernhall B (2010). Exercise and type 2 diabetes: American College of Sports Medicine and the American Diabetes Association: joint position statement. Exercise and type 2 diabetes. Med Sci Sports Exerc.

[25] Shirai M, Schwenke DO, Tsuchimochi H, Umetani K, Yagi N, Pearson JT (2013). Synchrotron radiation imaging for advancing our understanding of cardiovascular function. Circ Res.

[26] Figueiredo G, Brockmann C, Boll H, Heilmann M, Schambach SJ, Fiebig T (2012). Comparison of digital subtraction angiography, micro-computed tomography angiography and magnetic resonance angiography in the assessment of the cerebrovascular system in live mice. Clin Neuroradiol.

[27] Makino A, Ohuchi K, Kamata K (2000). Mechanisms underlying the attenuation of endothelium-dependent vasodilatation in the mesenteric arterial bed of the streptozotocin-induced diabetic rat. Br J Pharmacol.

[28] Lee S, Park Y, Zhang C (2011). Exercise training prevents coronary endothelial dysfunction in type 2 diabetic mice. Am J Biomed Sci.

[29] Kuru O, Senturk UK, Kocer G, Ozdem S, Baskurt OK, Cetin A (2009). Effect of exercise training on resistance arteries in rats with chronic NOS inhibition. J Appl Physiol.

[30] McAllister RM, Jasperse JL, Laughlin MH (2005). Nonuniform effects of endurance exercise training on vasodilation in rat skeletal muscle. J Appl Physiol.

[31] McAllister RM, Newcomer SC, Laughlin MH (2008). Vascular nitric oxide: effects of exercise training in animals. Appl Physiol Nutr Metab.

[32] Zguira MS, Vincent S, Le Douairon Lahaye S, Malarde L, Tabka Z, Saiag B (2013). Intense exercise training is not effective to restore the endothelial NO-dependent relaxation in STZ-diabetic rat aorta. Cardiovasc Diabetol.

[33] Kindig CA, Sexton WL, Fedde MR, Poole DC (1998). Skeletal muscle microcirculatory structure and hemodynamics in diabetes. Respir Physiol.

[34] Wang CH, Chen KT, Mei HF, Lee JF, Cherng WJ, Lin SJ (2011). Assessment of mouse hind limb endothelial function by measuring femoral artery blood flow responses. J Vasc Surg.

[35] Copp SW, Hageman KS, Behnke BJ, Poole DC, Musch TI (2010). Effects of type II diabetes on exercising skeletal muscle blood flow in the rat. J Appl Physiol.

[36] Chimen M, Kennedy A, Nirantharakumar K, Pang TT, Andrews R, Narendran P (2012). What are the health benefits of physical activity in type 1 diabetes mellitus? A literature review. Diabetologia.

[37] Huang HH, Farmer K, Windscheffel J, Yost K, Power M, Wright DE (2011). Exercise increases insulin content and basal secretion in pancreatic islets in type 1 diabetic mice. Exp Diabetes Res.

[38] Paulson DJ, Mathews R, Bowman J, Zhao J (1992). Metabolic effects of treadmill exercise training on the diabetic heart. J Appl Physiol.

[39] Selagzi H, Buyukakilli B, Cimen B, Yilmaz N, Erdogan S (2008). Protective and therapeutic effects of swimming exercise training on diabetic peripheral neuropathy of streptozotocin-induced diabetic rats. J Endocrinol Invest.

[40] Wallberg-Henriksson H, Gunnarsson R, Rossner S, Wahren J (1986). Long-term physical training in female type 1 (insulin-dependent) diabetic patients: absence of significant effect on glycaemic control and lipoprotein levels. Diabetologia.

